# The complete chloroplast genome sequence of medicinal plant, *Artemisia Montana*

**DOI:** 10.1080/23802359.2020.1768941

**Published:** 2020-05-20

**Authors:** Xueyun Cao, Miao Zhao, Jing Qi

**Affiliations:** Zhang Zhongjing School of Chinese Medicine, Nanyang Institute of Technology, Nanyang, PR China

**Keywords:** *A. montana*, chloroplast genome, phylogenetic analysis, genetic information

## Abstract

The complete chloroplast genome sequence of *Artemisia montana* was characterized from Illumina pair-end sequencing. The chloroplast genome of *A. montana* was 151,130 bp in length, containing a large single-copy region (LSC) of 80,975 bp, a small single-copy region (SSC) of 16,011 bp, and two inverted repeat (IR) regions of 27,162 bp. The overall GC content is 30.70%, while the correponding values of the LSC, SSC, and IR regions are 64.6%, 69.2%, and 60.1%, respectively. The genome contains 131 complete genes, including 86 protein-coding genes (62 protein-coding gene species), 37 tRNA genes (29 tRNA species) and 8 rRNA genes (4 rRNA species). The Neighbour-joining phylogenetic analysis showed that *A. montana* and *Artemisia lavandulaefolia YC* clustered together as sisters to other *Artemisia* species.

## Introduction

*Artemisia montana* is a perennial herb as a member of the Artemisia belonged to the family Asteraceae and widely distributed in China, which has persisted largely in an undomesticated state that is highly resistant to different environmental stresses (Hou et al. 2018). *A. montana* has high ecological and economic value with high levels of intraspecific genetic diversity. *A. montana* has wide geographic distribution, high intraspecific polymorphism, adaptability to different environments, combined with a relatively small genome size. Consequently, *A. montana* represents an excellent model for understanding how different evolutionary forces have sculpted the variation patterns in the genome during the process of population differentiation and ecological speciation (Neale and Antoine 2011). Moreover, we can develop conservation strategies easily when we understand the genetic information of *A. montana*. In the present research, we constructed the whole chloroplast genome of *A. montana* and understood many genome varition information about the species, which will provide beneficial help for population genetics studies of *A. montana*

The fresh leaves of *A. montana* were collected from Nanchong (106°08′E; 30°78′N). Fresh leaves were silica-dried and taken to the laboratory until DNA extraction. The voucher specimen (SDH001) was laid in the Herbarium of Nanyang Institute of Technology and the extracted DNA was stored in the −80 °C refrigerator of the Key Laboratory of School of Biological and Chemical Engineering. We extracted total genomic DNA from 25 mg silica-gel-dried leaf using a modified CTAB method (Doyle 1987). The whole-genome sequencing was then conducted by Biodata Biotechnologies Inc. (Hefei, China) with Illumina Hiseq platform. The Illumina HiSeq 2000 platform (Illumina,San Diego, CA) was used to perform the genome sequence. We used the software MITObim 1.8 (Hahn et al. 2013) and metaSPAdes (Nurk et al. 2017) to assemble chloroplast genomes. We used *P. tremula* (GenBank: NC_027425) as a reference genome. We annotated the chloroplast genome with the software DOGMA (Wyman et al. 2004), and then corrected the results using Geneious 8.0.2 (Campos et al. 2016) and Sequin 15.50 (http://www.ncbi.nlm.nih.gov/Sequin/).

The complete chloroplast genome of *A. montana* (GenBank accession number NC025910) was characterized from Illumina pair-end sequencing. The complete chloroplast genome sequence of *A. montana* was characterized from Illumina pair-end sequencing. The complete chloroplast genome sequence of *Artemisia montana* was characterized from Illumina pair-end sequencing. The chloroplast genome of *A. montana* was 151,130 bp in length, containing a large single-copy region (LSC) of 80,975 bp, a small single-copy region (SSC) of 16,011 bp, and two inverted repeat (IR) regions of 27,162 bp. The overall GC content is 30.70%, while the correponding values of the LSC, SSC, and IR regions are 64.6%, 69.2%, and 60.1%, respectively. The genome contains 131 complete genes, including 86 protein-coding genes (62 protein-coding gene species), 37 tRNA genes (29 tRNA species) and 8 rRNA genes (4 rRNA species).

**Figure 1.**
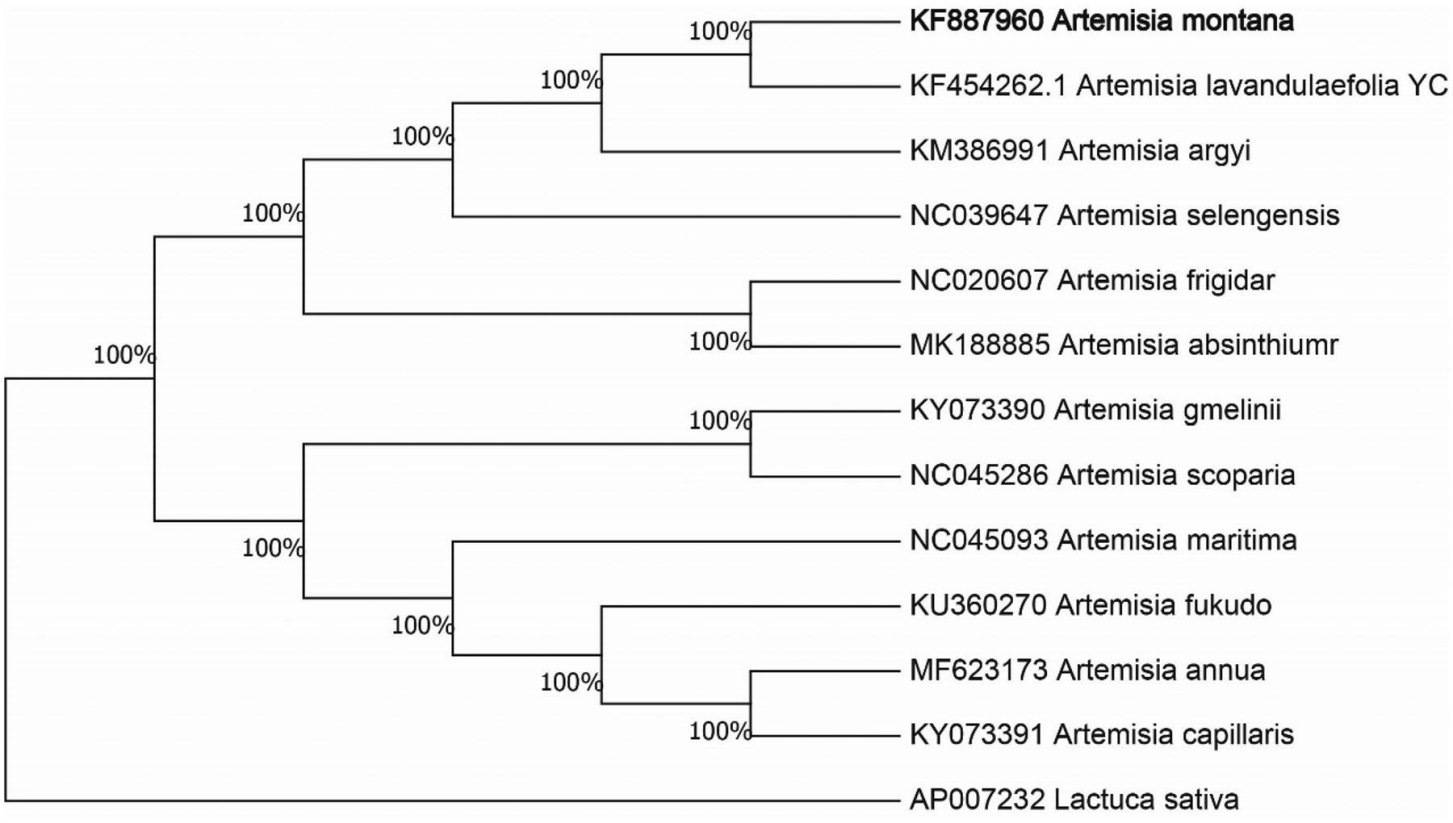
Neighbour-joining (NJ) analysis of A. gmelinii and other related species based on the complete chloroplast genome sequence. *Lactuca sativa* (AP007232) was set as the outgroup. All other sequences were downloaded from NCBI GenBank.

We used the complete chloroplast genomes sequence of *A. montana* and 11 other related species of *Artemisia* and *Lactuca* sativa as outgroup to construct phylogenetic tree. The 13 chloroplast genome sequences were aligned with MAFFT (Katoh and Standley 2013), and then the Neighbour-joining tree was constructed by MEGA 7.0 (Kumar et al. 2016). The results confirmed that *A. montana* was clustered with *Artemisia lavandulaefolia YC* (Figure 1).

## Data Availability

The data that support the findings of this study are openly available in GenBank at https://www.ncbi.nlm.nih.gov/nuccore/NC_025910.1, reference number NC025910.1.
